# Comprehensive Analysis of *Miscanthus* NF-YA Genes Reveals Potential Involvement in Drought Stress Adaptation

**DOI:** 10.3390/plants14193100

**Published:** 2025-10-08

**Authors:** Yang Yu, Mengting Li, Ming Yu, Tingting Wang

**Affiliations:** 1 Tianjin Crop Research Institute, Tianjin Academy of Agricultural Sciences, Tianjin 300192, China; 2 College of Agronomy, Northwest Agriculture and Forestry University, Xianyang 712100, China; nwafuyuming@163.com (M.Y.); wtt747831@163.com (T.W.); 3 Agro-Environmental Protection Institute, Ministry of Agriculture and Rural Affairs, Tianjin 300191, China; 4 College of Food Science and Bioengineering, Tianjin Agricultural University, Tianjin 300384, China

**Keywords:** *Miscanthus*, drought stress, MsNF-YA4, transgenic plants

## Abstract

*Miscanthus*, a perennial grass, is renowned for its remarkable tolerance to abiotic stress. Excessive levels of drought severely impair plant growth and yield. Plant nuclear factor Y (NF-Y) transcription factors (TFs) play pivotal roles in regulating responses to drought stress in species such as *Arabidopsis* and maize. However, their functional roles in conferring drought tolerance in *Miscanthus* remain largely unexplored. This study’s genome-wide analysis and gene expression profiling of *Miscanthus* under dehydration/osmotic stress identified a transcription factors gene, *MsNF-YA4*, which was significantly upregulated under dehydration/osmotic stress. *MsNF-YA4* overexpression in *Arabidopsis* significantly enhanced drought tolerance, leading to increased transcription of stress- and antioxidant enzyme-related genes. Compared with the wild type (WT), the transgenic lines exhibited markedly higher relative water content (RWC), chlorophyll content, proline level, and antioxidant enzyme activity. Furthermore, the MsNF-YA4/MsNF-YB3/MsNF-YC2 improved the transactivation of the *Miscanthus P5CS1*, *SOD* (Cu/Zn) and *CAT1* promoters in the transient system. These results offer fresh perspectives on the role of *Miscanthus* NF-YAs in drought tolerance and offer promising genetic resources for developing drought-tolerant crops through breeding programs.

## 1. Introduction

Global plant growth and crop yields are adversely hindered by abiotic stresses, namely drought, salinity, and heavy metals. Transcription factors (TFs) are proteins that regulate gene expression by binding to the promoter DNA sequences of target genes. Their activity is modulated by various signals and plays a crucial role in processes such as plant development and stress responses [1,2]. Under abiotic stress, many stress-response TFs initiate transcription cascades that regulate the transcriptional levels of target genes, enhance physiological characteristics, and alleviate stress [3]. NF-Y members, which target the CCAAT motif, comprise a heterotrimeric assembly of NF-YA/B/C subunits and have been widely explored in diverse living organisms [4,5]. This structural feature implies that the transactivation activity observed in transient assays may reflect cooperative interactions among the subunits, rather than the effect of a single NF-Y member alone [5]. The NF-YB/C subunits form heterodimers in the cytoplasm through a histone-fold motif, which then combine with NF-YA in the nucleus to create a heterotrimer that binds to specific target gene elements and facilitates permissive chromatin modifications [6,7,8]. Research indicates the expression of plant NF-YA members, revealing their diverse functions [9]. NF-YA TFs are involved in flavonoid biosynthesis [10], root developmental changes [11,12], leaf growth [13], flowering regulation [14,15,16], juvenile-to-adult transition of plants [17], plant disease resistance [18], starch accumulation [19], and secondary cell wall development [20]. Some NF-Ys are crucial regulators of environmental stress responses to drought, salt, low temperatures, and heavy metals. Overexpression of NF-Y family members enhances plant resilience to drought (e.g., *SbNF-YA6*, *PbYA4a*, *ZmNF-YB10*, *ZmNF-YC12*), salt stress (e.g., *GmNF-YC14*, *SiNF-YC2*, *GmNF-YA14*, *OsNFYA2*), and cold stress (e.g., *NF-YA2/7/10*, *AtHAP5A*, NF-Y (YZ9) [21,22,23,24,25,26,27,28,29,30,31]. NF-Y proteins regulate heavy metal stress adaptations in plants. For example, I1NF-YC6 enhances plant tolerance to Cd by mediating *IlCDT1* transcription [32], whereas AtNF-YC3 interacts with CIPK21 to regulate the peroxidase system, scavenging Cd stress, and inducing reactive oxygen species (ROS), enhancing *Arabidopsis* tolerance to Cd stress [33]. These studies underscore the potential of NF-Y TFs in crop engineering to improve abiotic stress. In plants, the number of NF-YA members may vary across genome versions; for instance, ten to eleven NF-YAs have been reported in rice depending on the annotation [34,35]. Genome-wide studies have identified NF-YA members in species such as peaches [36], Populus [37], peanuts [38], Chinese cabbage [39], apple [16], rice [35], and tobacco [40]. However, the NF-YAs are yet to be investigated in *Miscanthus* under abiotic stress, leaving the roles and regulatory mechanisms of the NF-YA members in abiotic stress, especially in response to drought stress, largely unknown.

*Miscanthus*, a C_4_ perennial grass from the *Poaceae* family native to East Asia, thrives across a broad geographic and climatic range, including Asia, Siberia, and both southern and northern Europe, and is characterized by a strong ability for vegetative propagation, having initially been valued for its aesthetic appeal in landscape design [41]. In recent years, the cultivation and production of *Miscanthus* have been expanding due to its high sustainability and extensive applications in environmental remediation, renewable energy, and industrial bioprocessing [42,43,44]. Thriving on marginal lands and buffer strips without competing for croplands, it requires minimal agrotechnical inputs while maintaining high yields over multiple years [44]. As a C_4_ plant, it enhances carbon sequestration and significantly reduces CO_2_ emissions [45]. Additionally, its ability to remediate soil contamination, including heavy metals and oil spills, further underscores its ecological value. As an energy crop, *Miscanthus* can help reduce dependence on forest resources. For example, its use as a biomass fuel and fiber source decreases the demand for wood, thereby contributing to forest conservation. Compared to traditional timber, *Miscanthus* exhibits a shorter growth cycle, offering a more rapidly renewable energy source [46]. Beyond environmental benefits, *Miscanthus* serves as a versatile resource in sustainable energy production [47,48]. It can be used directly as fuel pellets and biocoal or converted into bioethanol and biogas, contributing to a reduced carbon footprint [49]. Its biomass, rich in cellulose, also holds significant industrial potential: it enhances energy efficiency in construction as an insulation material, serves as a feedstock for cellulose extraction and functionalization, and undergoes fractionation to yield high-value chemicals such as acetic acid, glycolaldehyde, and acetol [50]. Additionally, hydrolysis of its cellulose component produces carbohydrate-rich substrates essential for enzyme biosynthesis, bacterial nanocellulose production, and bioethanol generation [41]. These multifaceted applications position *Miscanthus* as a key driver of sustainable development, integrating ecological restoration with industrial innovation. Comprising approximately 17 distinct species, *Miscanthus* displays complex genomic diversity, which enhances its adaptability to various environmental conditions. To date, genomic information from at least four species of *Miscanthus* has been released (*M. sinensis*, *M. sacchariflorus*, *M. floridulus*, and *M. lutarioriparius*), increasing the possibility of genetic improvement of *Miscanthus* [51]. *M. sinensis*, *M. floridulus*, *M. sacchariflorus*, and *M. lutarioriparius* are the primary species within the *Miscanthus* genus, predominantly distributed across Asia [44,52,53]. Among them, *M. sinensis* and *M. sacchariflorus* have been the most extensively studied due to its broader natural distribution and superior environmental adaptability compared to other *Miscanthus* species [51,53]. *M. floridulus*, a major *Miscanthus* species in southern China, has limited potential as a bioenergy crop due to its low cold tolerance and non-natural wilting characteristics. Studies have shown that *M. floridulus* may have evolved from *M. sinensis* [52]. *M. lutarioriparius*, an endemic *Miscanthus* species in the Dongting Lake region of the middle and lower reaches of the Yangtze River in China, could grow up to 7 m in height and exhibits a higher photosynthetic rate than other *Miscanthus* species [53,54].

Studies on the regulation of *Miscanthus NF-YA* genes under drought stress are limited, and their drought-responsive regulatory network remains unclear. In this study, we identified *Miscanthus* MsNF-YA4, an NF-Y transcription factor, as a key regulator of drought stress tolerance. Our findings revealed that MsNF-YA4 activates downstream stress-associated genes. The investigation was organized around two principal objectives: first, to demonstrate that MsNF-YA4 functions as a central regulator of drought stress tolerance; and second, to elucidate the role of MsNF-YA4 in facilitating the activation of stress-responsive genes. This study offers new insights into the *MsNF-YA* gene and highlights the pivotal role of MsNF-YA4 in mediating drought tolerance in *Miscanthus*.

## 2. Results

### 2.1. Characterization and Phylogenetic Relationship Assessment of Miscanthus NF-YAs

**Figure 1:**
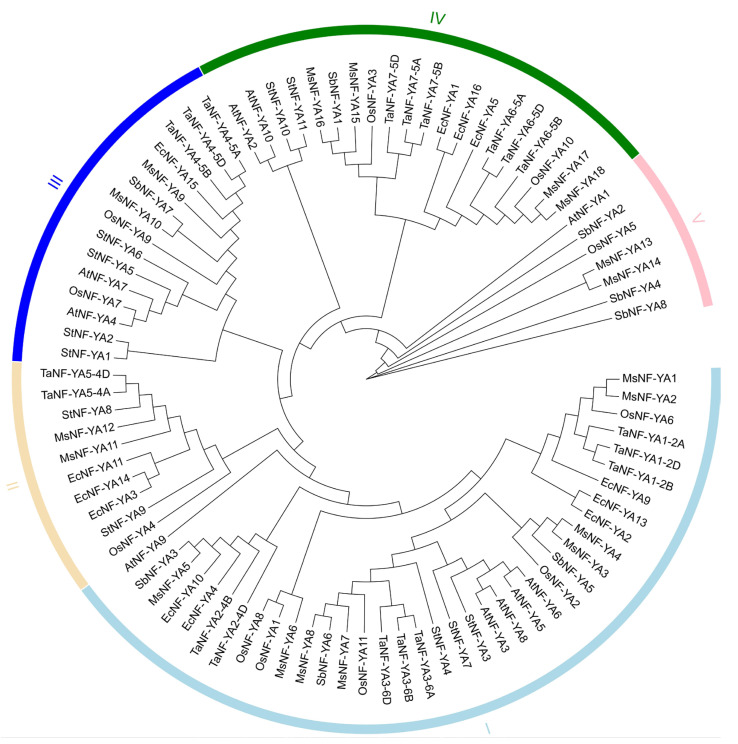
Phylogenetic relationships of NF-YA proteins. The maximum likelihood (ML) method was applied in MEGA7 to construct a phylogenetic tree of NF-YAs in *Arabidopsis*, rice, wheat, finger millet, sorghum, potato and *Miscanthus*.

Using the Phytozome, and NCBI-CDD databases, we identified 18 proteins containing NF-YA domains in *Miscanthus*. These proteins were designated MsNF-YA1 through MsNF-YA18 based on their phylogenetic relationships (Figure 1). The 18 *MsNF-YA* genes were scattered unevenly across eight different chromosomes: four MsNF-YAs on each chromosomes 01 and 02; two members on chromosomes 14 and 15 each, one gene on chromosomes 04, 07, and 08; and three genes on chromosome 03 (Figure S1; Table S1). The size, pI, MW, and subcellular localization of the 18 NF-Ys were determined and predicted (Table S1). The protein sizes of MsNF-YAs differed significantly, ranging from 197 AA (MsNF-YA13) to 385 AA (MsNF-YA18). Their MWs ranged from 21.36 kDa (MsNF-YA13) to 41.81 kDa (MsNF-YA18), and pIs ranged from 7.74 (MsNF-YA4) to 11.43 (MsNF-YA8). Notably, all NF-YA proteins in *Miscanthus* are predominantly localized in the nucleus.

A phylogenetic tree was generated to examine the evolutionary relationships of NF-YA members in *Miscanthus*, incorporating NF-YA proteins from 10 *Arabidopsis*, 11 rice, 16 finger millet, 8 sorghum, 11 potato, 18 *Miscanthus*, and 19 wheat sequences (Figure 1). The genome sizes of *Arabidopsis*, rice, wheat, finger millet, sorghum, potato, and *Miscanthus* are 135 Mb, 500 Mb, 17 Gb, 2.08 Gb, and 1.11 Gb, respectively, based on data obtained from the EnsemblPlants and Phytozome databases. Among them, common wheat, an allopolyploid with a complex hexaploid genome, contains a relatively large number of TaNF-YA members (19). In contrast, sorghum has fewer SbNF-YA members (8). All NF-YA proteins in *Miscanthus* are categorized into five Groups: I–V. Groups I, II, III, IV and V contained eight (MsNF-YA1−8), two (MsNF-YA11−12), two (MsNF-YA9−10), four (MsNF-YA15−18), and two (MsNF-YA13−14) MsNF-YA members, respectively.

### 2.2. Exon–Intron Structure and Protein-Motif Analysis of MsNF-YAs

**Figure 2:**
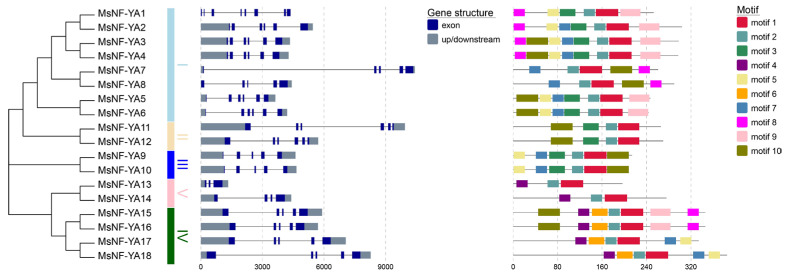
Gene structures and conserved motifs of NF-YA members in *Miscanthus*.

The gene structure of the MsNF-YAs varied dramatically (Figure 2). The quantity of introns and exons in *Miscanthus* NF-YAs varied from two to seven and three to eight, respectively, with *MsNF*-*YA13* having the lowest number of introns and exons (two and three, respectively), whereas *MsNF*-*YA1* had the highest number (seven and eight, respectively).

**Figure 3:**
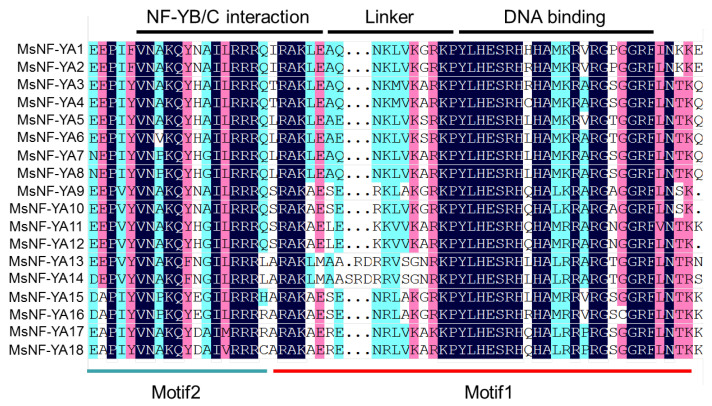
Multiple alignments and predicted structure of conserved regions of NF-YA members in *Miscanthus*.

Multiple alignments of MsNF-YA proteins showed that the conserved central region of MsNF-YA consists of two subdomains: One responsible for mediating NF-YB/C interactions and the other for binding to the CCAAT-box (Figure 3). In total, 10 protein motifs in the MsNF-Yas in *Miscanthus* were identified using MEME (v. 5.5.6), and their logos are shown in Figure S2. Motifs 1/2, which encode the NF-YB/C interaction and DNA-binding regions, were present in all 18 MsNF-YA members (Figure 2 and Figure 3), indicating that motifs 1 and 2 play a central role in maintaining NF-YA function compared with other motifs.

### 2.3. Synteny and Cis-Regulatory Element Analysis of MsNF-YA Members in Miscanthus

**Figure 4:**
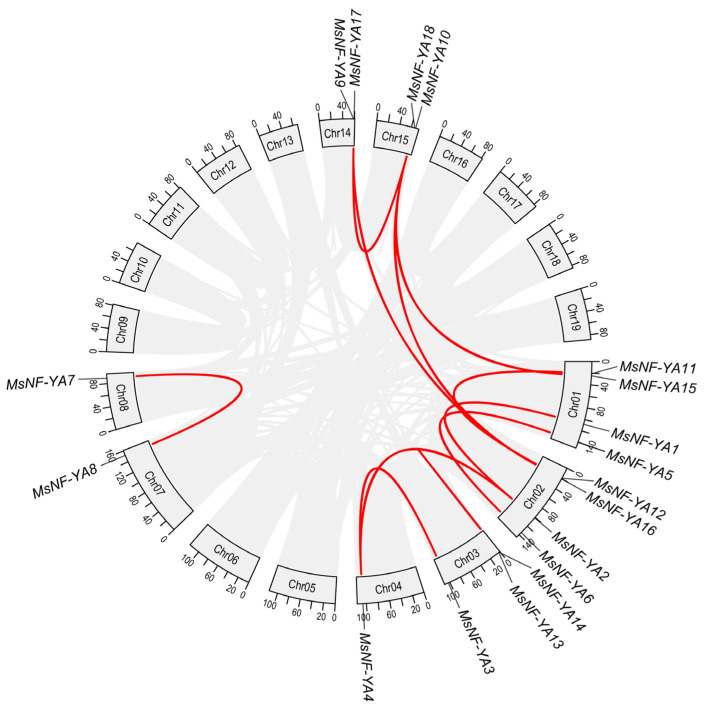
The collinear relations of NF-YA genes in the *Miscanthus* genome.

In total, 11 duplicated gene pairs were identified in *Miscanthus NF*-*YA*, including one tandem duplication pair (9.09%) and ten segmental duplication pairs (90.91%) (Figure 4; Table S2). These results suggest that some MsNF-YAs may have originated from gene replication events, with segmental replication being the dominant mode of NF-YA gene evolution. The Ka/Ks ratios for NF-YA genes in *Miscanthus* varied from 0.25 to 1.02, with a mean of 0.49 (Table S2), implying that these genes have experienced purifying selection.

**Figure 5:**
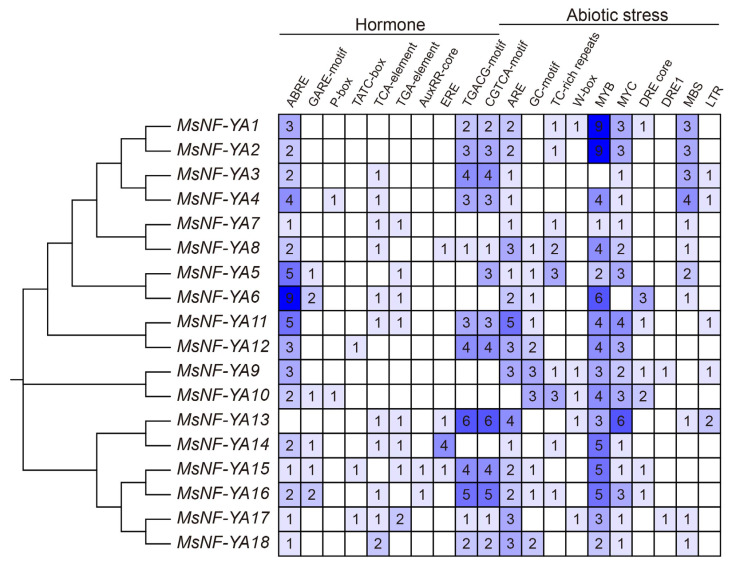
Predicted *cis*-acting elements of *Miscanthus NF*-*YA* genes. The hormone- and abiotic stress-associated *cis*-acting elements of 2 kb promoter region of *NF*-*YA* genes were identified in *Miscanthus*.

To investigate the potential roles of the MsNF-YAs, 32 *cis*-acting elements within their promoters were characterized using PlantCARE. These elements were associated with hormonal and environmental stress factors (Figure 5). 12 phytohormone response elements were identified, including ABA (ABREs), gibberellin (P-box, TATC-box, and GARE motifs), salicylic acid (TCA elements), auxin (AuxRR core and TGA element), ethylene (EREs), and MeJA elements (CGTCA and TGACG motifs). Moreover, 20 abiotic stress-responsive elements were found in MsNF-YA genes, including hypoxia induction (GC-motif), anaerobic induction (ARE), defense and stress responses (TC-rich repeats A), WRKY protein-binding element (W-box), MYB/MYC factor-binding elements (MYB/MYC), dehydration-responsive element (DRE core, DRE1), drought induction (MBS), NF-Y factor binding element (CCAAT-box), and cold response element (LTR). These results indicated that MsNF-YA factors are involved in hormonal reactions and regulate multiple abiotic stress responses.

### 2.4. Effects of Dehydration/Osmotic Stress on the Relative Water Content (RWC) in Miscanthus

RWC is regarded as a measure of plant water status, reflecting the metabolic activity of the plant organization. It is usually used as an index of dehydration in most plants. As shown in Figure S3, the RWC of leaves exhibited a continuous decline with prolonged stress duration. At 0 h, RWC was approximately 93%, representing a well-hydrated state. After 6 h, it decreased significantly to about 90%, followed by further reductions to 86% at 12 h, 80% at 24 h, and 77% at 48 h. These findings demonstrate that leaf RWC decreases progressively under PEG-induced dehydration/osmotic stress, indicating increasing water deficit and disruption of cellular water balance.

### 2.5. Investigation of Miscanthus NF-YA Members Under Dehydration/Osmotic Stress

**Figure 6:**
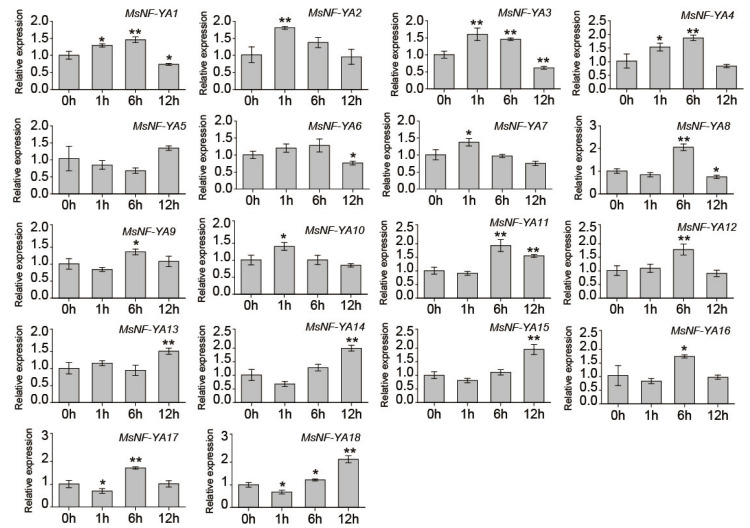
Gene expression levels of *NF*-*YA* genes in *Miscanthus* leaves under dehydration/osmotic stress. Error bars show standard deviations (mean ± SD and n = 3) and significant variations are indicated by asterisks (Student’s *t*-test, *p* < 0.05 (*); *p* < 0.01 (**)).

To further explore the function of *Miscanthus* NF-YAs under dehydration/osmotic stress, we assessed their expression profiles using qRT-PCR (Figure 6). The results revealed that most *MsNF-YA* genes responded to dehydration/osmotic stress, exhibiting distinct expression patterns over time. Several genes, including *MsNF-YA1*, *MsNF-YA2*, *MsNF-YA4*, *MsNF-YA7*, and *MsNF-YA10*, were rapidly induced at 1 h. Many genes, such as *MsNF-YA3*, *MsNF-YA4*, *MsNF-YA8*, *MsNF-YA9*, *MsNF-YA11*, *MsNF-YA12*, *MsNF-YA16*, *MsNF-YA17*, and *MsNF-YA18*, showed peak expression levels at 6 h. By 12 h, the expression of some genes, such as *MsNF-YA1* and *MsNF-YA3*, began to decline, whereas others, including *MsNF-YA11* and *MsNF-YA18*, remained highly expressed. In contrast, MsNF-YA5 exhibited little-to-no change throughout the entire time course. These findings suggest that some *MsNF-YA* genes respond to dehydration/osmotic stress and may regulate plants’ response to drought stress.

### 2.6. Functional Analysis of MsNF-YA4 Overexpression in Arabidopsis Under Drought Stress

**Figure 7:**
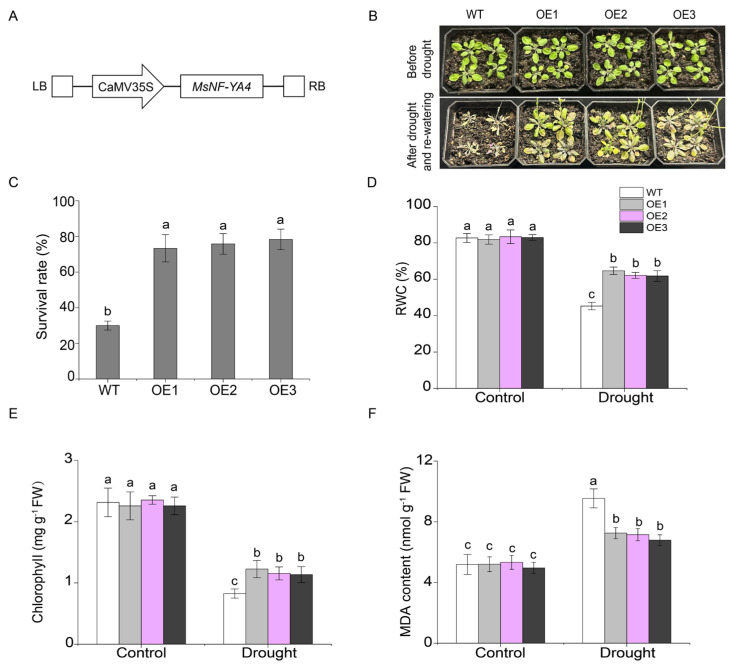
Functional analysis of *MsNF-YA4* transgenic *Arabidopsis* under drought stress. (**A**) Schematic representation of the pBI121 plant overexpression vector carrying the *MsNF-YA4* gene under the control of the CaMV35S promoter. (**B**) Photographs were taken before drought stress, 13 days for withholding water or after 3 days for recovery period. The survival rate (**C**), RWC (**D**), chlorophyll (**E**), and MDA (**F**) were measured. The data are presented as mean ± SD (n = 3), with differences analyzed by one-way ANOVA followed by Tukey’s test (*p* < 0.05), and different letters indicate significant differences.

**Figure 8:**
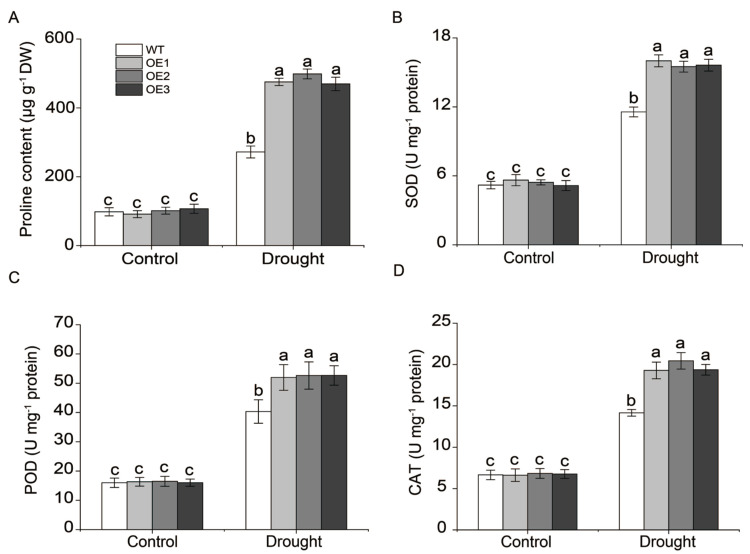
Four physiological indicators were measured under drought stress: proline (**A**), and the activities of SOD (**B**), POD (**C**), and CAT (**D**) antioxidant enzymes. The data are presented as mean ± SD (n = 3), with differences analyzed by one-way ANOVA followed by Tukey’s test (*p* < 0.05), and different letters indicate significant differences.

Under drought stress, among the 18 identified *MsNF-YA* genes, *MsNF-YA4* expression gradually increased at 1 h, reaching a peak at 6 h followed by a decline towards the baseline at 12 h (Figure 6). Additionally, *cis*-element analysis of the *Miscanthus NF-YA4* promoter revealed the presence of various elements associated with abiotic stress response (Figure 5). Therefore, this gene was selected for further functional analysis. To explore its function, a *MsNF-YA4* overexpression vector (Figure 7A) was generated and introduced into *Arabidopsis* through *Agrobacterium*-mediated floral dip transformation, resulting in the development of homozygous T_3_ transgenic lines. The expression levels of *MsNF-YA4* in the OE *Arabidopsis* plants were measured using qRT-PCR (Figure S4). All ten OE lines exhibited marked elevation of *MsNF-YA4* expression relative to the control, particularly in OE1, OE2, and OE3. RT-PCR analysis verified that *MsNF-YA4* expression was solely present in overexpression (OE) plants, with no detectable expression in wild-type (WT) plants, indicating the successful transformation of *MsNF-YA4* into *Arabidopsis* (Figure S5). After 13 days of drought followed by 3 days of re-watering, the survival rates of the transgenic OE lines were 73.33–78.33%, significantly higher than the 30% observed in WT plants (Figure 7C). Under drought stress, the WT plants exhibited more pronounced wilting and impaired growth. In contrast, the OE lines showed relatively milder symptoms (Figure 7B). Further physiological analyses were performed to assess the effects of *MsNF-YA4* overexpression. Under normal watering conditions, the WT and OE lines showed no significant differences in RWC (Figure 7D), chlorophyll (Figure 7E), MDA content (Figure 7F), proline content (Figure 8A), SOD (Figure 8B), POD (Figure 8C), and CAT (Figure 8D). However, under drought stress, most physiological parameters showed an increasing trend in both WT and OE lines, although the patterns differed. In the OE lines, chlorophyll, and proline content, as well as the activities of POD, SOD, and CAT, were markedly elevated. In contrast, the MDA level was considerably lower than that in the WT plants.

**Figure 9:**
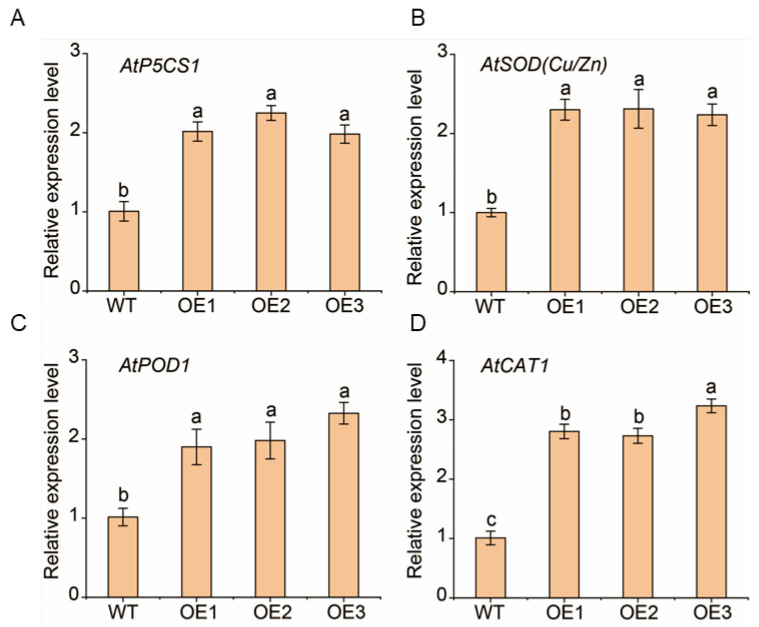
Expression levels of stress-related genes under drought stress. Four genes were measured: *AtP5CS1* (**A**), *AtSOD* (Cu/Zn) (**B**), *AtPOD1* (**C**), and *AtCAT1* (**D**). The data are presented as mean ± SD (n = 3), with differences analyzed by one-way ANOVA followed by Tukey’s test (*p* < 0.05), and different letters indicate significant differences.

To explore the possible regulatory mechanism of *MsNF-YA4* under drought stress, we analyzed stress- (*AtP5CS1*) and antioxidant-associated genes (*AtSOD* (Cu/Zn), *AtPOD1*, and *AtCAT1*) in *Arabidopsis* for further investigation (Figure 9). Under drought stress, the transcript levels of these genes were more elevated in the *MsNF-YA4* OE lines than in the WT plants, indicating that overexpression of *MsNF-YA4* in *Arabidopsis* may enhance drought tolerance by modulating the transcript levels of these genes, possibly affecting the associated variations in physiological traits and provide a positive regulatory effect on drought stress responses.

### 2.7. The Regulatory Role of the MsNF-YA4/MsNF-YB3/MsNF-YC2 Module on MsP5CS1, MsSOD (Cu/Zn), MsPOD1, and MsCAT1

We predicted potential proteins interacting with MsNF-YA4 using the STRING database (based on the *Arabidopsis* model). This analysis identified seven NF-YB proteins (NF-YB1/B2/B3/B5/B6/B7) and four NF-YC (NF-YC1/C2/C3/C9) proteins (Table S3; Figure S6). Subsequent BLAST (Phytozome *Miscanthus sinensis* v7.1) comparisons identified seven NF-YB proteins (MsNF-YB1, MsNF-YB2, MsNF-YB3, MsNF-YB5, MsNF-YB6, and MsNF-YB7) and four NF-YC proteins (MsNF-YC1, MsNF-YC2, MsNF-YC3, and MsNF-YC9) in *Miscanthus* (Table S3). Yeast transformants containing recombinant vectors BD-MsNF-YA4/AD-MsNF-YB1, BD-MsNF-YA4/AD-MsNF-YB2, BD-MsNF-YA4/AD-MsNF-YB3, BD-MsNF-YA4/AD-MsNF-YB5, BD-MsNF-YA4/AD-MsNF-YB6, BD-MsNF-YA4/AD-MsNF-YB7, BD-MsNF-YA4/AD-MsNF-YC1, BD-MsNF-YA4/AD-MsNF-YC2, BD-MsNF-YA4/AD-MsNF-YC3, and BD-MsNF-YA4/AD-MsNF-YC9 were cultured on DDO and QDO/X media. These results indicate that MsNF-YA interacts with both MsNF-YB3 and MsNF-YC2 (Figure S7).

**Figure 10:**
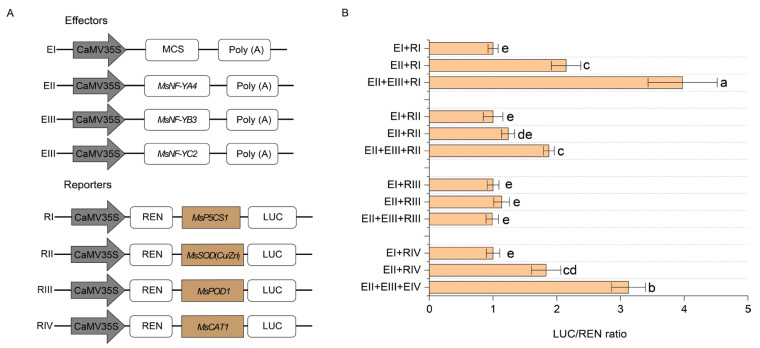
Dual-luciferase assay. (**A**) The reporter and effector constructs utilized in co-transfection are shown in a schematic diagram. (**B**) The transactivation effect of MsNF-YA4/MsNF-YB3/MsNF-YC2 on the promoter of *MsP5CS1*, *MsSOD* (Cu/Zn), *MsPOD1*, and *MsCAT1*. The data are presented as mean ± SD (n = 3), and differences were analyzed by one-way ANOVA with Tukey’s test (*p* < 0.05). Different letters indicate significant differences.

The expression levels of *AtP5CS1*, *AtSOD* (Cu/Zn), *AtPOD1*, and *AtCAT1* increased in *MsNF-YA4* overexpression lines, suggesting that MsNF-YA4 may downstream regulate these genes. We aimed to investigate whether MsNF-YA4 directly controls the expression of *MsP5CS1*, *MsSOD* (Cu/Zn), *MsPOD1*, and *MsCAT1* (homologous genes from *Arabidopsis* in *Miscanthus*). We conducted an analysis of their 2000 bp promoter sequence through PlantCARE. The results identified potential binding site motifs (CCAAT-box) in the promoters of these genes (Figure S8). Specifically, the promoters of *MsP5CS1*, *MsSOD* (Cu/Zn), *MsPOD1*, and *MsCAT1* gene contained three, two, one, and seven CCAAT box elements, respectively. Dual-luciferase assays showed a significant elevation in the LUC/REN ratio in plants carrying 35S:*MsNF-YA4*/Ms*P5CS1*:LUC and 35S:*MsNF-YA4*/MsCAT1:LUC compared with the control (Figure 10A,B). Plants harboring 35S: MsNF-YA4/MsNF-YB3/MsNF-YC2 and MsP5CS1/MsSOD (Cu/Zn)/MsCAT1:LUC exhibited an even larger increase in LUC/REN ratio (Figure 10B). However, plants harboring 35S:*MsNF-YA4* and *MsPOD1*:LUC or 35S:*MsNF-YA4*/*MsNF-YB3*/*MsNF-YC2* and *MsPOD1*:LUC exhibited no differences in the LUC/REN ratio. These results indicate that MsNF-YA can activate the promoter activity of *MsP5CS1* and *MsCAT1*; adding the other two interacting proteins further enhanced this capability. In summary, MsNF-YA4/MsNF-YB3/MsNF-YC2 may form a trimeric complex that positively regulates *MsP5CS1* and *MsCAT1*.

## 3. Discussion

Abiotic stress poses significant challenges to plant growth, adversely affecting development, yield, and quality [55]. Plants have evolved intricate physiological, biochemical, and molecular adaptations to survive and thrive under adverse environmental conditions [56,57]. These mechanisms are controlled through the gene expression control of stress-responsive genes that modulate plant physiological traits, mediating downstream phenotypic changes and ultimately enhancing stress resistance [55]. In plants, the NF-Y TF family plays crucial roles in stress response, photosynthesis, developmental and flowering time regulation, and hormone signaling integration [4,20,37]. By balancing growth and stress tolerance, NF-Y ensures plant growth and reproduction under challenging environmental conditions. *GhNF-YA* genes may modulate salt tolerance via ABA or MeJA signaling, and silencing GhNF-YA10 and GhNF-YA23 reduces salt tolerance in cotton [58]. Overexpression of *GmNFYA13* enhanced salt and drought tolerance in soybean, whereas its loss produced the opposite effect [59]. *TaNF-YA10–1* overexpression enhanced drought tolerance but increased plant sensitivity to salinity [60]. *TaNF-YA7* is highly responsive to PEG-induced dehydration and functions as a key regulator of drought adaptation by controlling stomatal movement, leaf water status, osmolyte accumulation, and ROS balance [61]. While the NF-Y family has been widely investigated in plants, investigations of *Miscanthus NF-Y* genes remain limited. The biological functions and regulatory mechanisms of NF-Y in *Miscanthus* remain unclear, and its role in response to drought stress merits further investigation. *Physcomitrella patens* is renowned for its exceptional stress tolerance, particularly its ability to survive in extreme environments, making it an important model organism for studying plant stress resistance mechanisms [62]. Studies have shown that heterologous overexpression of the *PpHSP70* gene from *Physcomitrella patens* in rice significantly enhances the heat and drought tolerance of transgenic rice [63]. Both *Bermudagrass* and *Miscanthus* belong to the *Poaceae* family of monocotyledonous plants, characterized by high abiotic-stress resilience [64,65]. Research has demonstrated that cloning and overexpressing the *NF-YC* gene from *Bermudagrass* significantly improves drought and salt tolerance in transgenic rice [66]. These findings suggest that using the stress-resistant *Miscanthus* as a research material for further exploration of the *Miscanthus NF-Y* gene family holds great potential for developing stress-resistant, high-yield crop varieties, providing valuable insights and applications for agricultural progress.

Plants undergo complex physiological and metabolic changes under abiotic stress (e.g., drought, salt, and heavy metal). Exposure to drought stress results in the generation of ROS, such as superoxide anions, hydrogen peroxide, and hydroxyl radicals, as a consequence of metabolic disturbances in the chloroplasts, mitochondria, and peroxisomes [67]. This interference impairs the electron transport chain, causing ROS leakage. Excessive ROS levels cause oxidative damage, including lipid peroxidation and the degradation of proteins and DNA [68]. As a by-product of lipid peroxidation, MDA is commonly employed to measure oxidative damage to cellular membranes [69]. To mitigate the impact of ROS, plants trigger both enzymatic and non-enzymatic antioxidant defense mechanisms, with elevated antioxidant enzyme activity enhancing ROS-scavenging efficiency [70,71]. Drought stress also inhibits the activity of chlorophyll-synthesis enzymes and accelerates chlorophyll degradation, leading to reduced chlorophyll content, decreased light-harvesting efficiency, and impaired photosynthesis, ultimately affecting plant growth [72]. In addition, proline, an osmoprotectant, accumulates significantly in response to stress. Proline functions mainly in osmotic regulation and in maintaining redox balance across different cell types rather than directly scavenging ROS [73]. Its primary roles include sustaining cellular osmotic balance (reducing water loss), stabilizing membranes, and indirectly mitigating drought-induced oxidative stress through improved intercellular balance [74]. In Figure 6, the expression of *MsNF-YA* genes was analyzed under PEG-induced osmotic stress. It should be noted that the experimental setup, in which plants were removed from soil, washed, and transferred to hydroponic culture, may itself have imposed additional stress (mechanical disturbance and ionic imbalance). Although most MsNF-YA genes clearly responded to PEG-induced dehydration/osmotic stress, the biological relevance of this treatment is limited. Under PEG-induced dehydration/osmotic stress, *MsNF-YA4* gene was significantly upregulated in *Miscanthus*. PEG treatment primarily imposes dehydration and osmotic stress at the cellular level, whereas soil or field drought involves a more complex physiological scenario, including water uptake limitations, root–soil interactions, and additional environmental variables [75,76]. Therefore, the results obtained under PEG-induced stress should be interpreted as a proxy for osmotic challenge, rather than as a direct equivalent of drought conditions in the soil or field. In this study, transgenic plants were grown in soil, and drought stress was simulated by withholding water. Under drought conditions, *MsNF-YA4* overexpressing plants exhibited higher survival rate, RWC, chlorophyll, and proline levels, as well as increased activities of antioxidant enzymes (SOD, POD, and CAT) compared with wild-type plants. Additionally, the transgenic lines displayed lower MDA levels than the wild-type lines. These results indicate that *MsNF-YA4* overexpressing lines possess enhanced drought tolerance, with improved photosynthetic performance, stronger antioxidant defense, better osmotic regulation (higher proline and RWC), and reduced oxidative damage (lower MDA) compared with wild-type plants. Beyond the transcriptional evidence obtained under PEG-induced dehydration/osmotic stress and the metabolic and antioxidant responses observed in Arabidopsis under PEG conditions, further insights into MsNF-YA4 function will require analyses of its gene localization and in situ expression dynamics during PEG stress. These studies are currently being planned and will help refine the mechanistic framework established in this study, providing a more comprehensive understanding of MsNF-YA4-mediated drought tolerance. The widespread occurrence of CCAAT motifs in plant promoters provides a rationale for analyzing putative *cis*-elements in NF-Y target genes [77]. However, it should be emphasized that in silico predictions of CCAAT-containing elements are only indicative, and experimental validation is required to confirm their functional relevance. In the present study, *MsP5CS1*, *MsSOD* (Cu/Zn), *MsPOD1*, and *MsCAT1* promoters contained MSNF-YA4-bound CCAAT-box elements, suggesting that MsNF-YA4 may affect the expression of stress-related genes. Luciferase assays demonstrate that MsNF-YA4 transactivates the promoter reporters of *MsP5CS1* and *MsCAT1*, leading to the upregulation of their transcription. NF-Y typically functions as a trimer (NF-YA/B/C), crucial for its transcriptional regulatory functions. Using yeast two-hybrid assay in this study, we identified two interacting proteins, MsNF-YB3 and MsNF-YC2. Interactions between the two proteins were also observed. Luciferase assays demonstrated that MsNF-YA4 activated the promoter activities of *MsP5CS1* and *MsCAT1* and that the addition of MsNF-YB3 and MsNF-YC2 further enhanced this activation in the transient system. This MsNF-YA4/MsNF-YB3/MsNF-YC2 transcriptional regulatory module may increase proline biosynthesis and antioxidant enzyme activity and alleviate drought stress by promoting osmotic homeostasis. Given the crucial role of *MsNF-YA4* under drought stress, it is an excellent genetic resource for breeding drought-tolerant crops. To further elucidate the role of MsNF-YA4 in drought tolerance beyond its functional characterization in *Miscanthus*, future studies will develop transgenic *Miscanthus* plants and employ a combination of transcriptome sequencing, ChIP-seq, and other techniques to uncover the molecular mechanisms underlying its regulatory functions.

## 4. Materials and Methods

### 4.1. Characterization and Phylogenetic Profiling of Miscanthus NF-YAs

The HMM profile (PF02045) was used as a query in HMMER 3.0 to search local protein libraries of *Miscanthus sinensis* genome version 7.1, which was downloaded from Phytozome on 21 January 2024. The resulting proteins were assessed on NCBI-CDD to further confirm the presence and structural integrity of the key domains. NF-YA protein sequences from *Arabidopsis*, rice, wheat, finger millet, sorghum, potato, and *Miscanthus* were processed through ClustalX for alignment. A phylogenetic tree was generated via the maximum likelihood approach in MEGA 7.0.

### 4.2. Plant Material, and Dehydration/Osmotic Treatments

*Miscanthus* plants were clonally propagated, transferred into potting soil with consistent plant size, and grown in a climate-controlled incubator at 25−28 °C with a 16 h light/8 h dark photoperiod [78]. At the 5–6 leaf stage, uniform *Miscanthus* plants were selected and exposed to PEG-induced dehydration/osmotic stress (20% PEG6000). For PEG stress treatment, plants were carefully removed from the soil and washed thoroughly. They were then submerged in a PEG solution [79]. The second fully expanded leaves from the top were harvested at 0, 1, 6, and 12 h after treatment, immediately frozen in liquid nitrogen, and stored at −80 °C.

### 4.3. RNA Extraction and qRT-PCR Analysis

Total RNA was isolated using the TianGen TRIzol reagent, and first-strand cDNA synthesis was carried out with the Transgen EasyScript^®^ First-Strand cDNA Synthesis SuperMix following the manufacturer’s instructions. The qRT-PCR was conducted using the Transgen SYBR^®^ Green I reaction kit on a Biosystems 7500 Fast Real-Time PCR System. Gene expression levels were determined using the 2^−ΔΔCT^ method, with *Miscanthus* internal reference gene (*Actin* (*Unigene33024*)). Previous studies have shown that 12 candidate reference genes were evaluated for their expression stability in Miscanthus under five different abiotic stress treatments (drought, salt, cadmium, chromium, and arsenic) using geNorm, NormFinder, BestKeeper, and RefFinder. Among them, *Unigene33024* was identified as the most stably expressed reference gene [80]. Each experiment was conducted with three biological replicates. Details of the primer sequences could be found in Table S4.

### 4.4. Characterization of NF-YA Genes and Proteins in Miscanthus

Gene structures were visualized using GSDS, and conserved motif analysis was performed via the MEME Suite. Gene annotations and protein sequences were obtained from the Phytozome.

### 4.5. Gene Duplication and Cis-Regulatory Element Analysis of Miscanthus NF-YAs

Gene duplication analysis was performed following the approach outlined by Yu et al. [81]. Syntenic relationships were visualized, and Ks and Ka substitution rates were estimated using TBtools (v.2.119). *Cis*-acting elements were detected by examining the 2000 bp upstream regions of the ATG in the NF-YAs of *Miscanthus* using PlantCARE [82]. It should be noted that *cis*-element prediction is based on sequence similarity and may include false positives, and the presence of predicted motifs does not necessarily imply functional activity in vivo.

### 4.6. Creation of Transgenic Arabidopsis Lines and Evaluation of Physiological Characteristics

The construction of plant overexpression vectors for *MsNF*-*YA4* transformation into *Arabidopsis* and the generation of T_3_ homozygous lines followed the previously described protocols [81]. The coding sequence (CDS) of *MsNF-YA4* was cloned into a modified pBI121 expression vector and introduced into wild-type *Arabidopsis thaliana* (Columbia-0) via the *Agrobacterium tumefaciens* strain GV3101 using the floral-dip method. Transgenic T_1_ seedlings were first screened on 1/2 MS medium supplemented with 50 mg L^−1^ kanamycin, then transferred to soil and grown to maturity to obtain T_2_ seeds. Individual T_2_ generation plants that segregated in 3:1 Mendelian ratio for kanamycin resistance were selected and homozygous T_3_ lines identified. In total, ten homozygous T_3_ transgenic lines were established. Three-week-old *Arabidopsis* plants, grown under controlled conditions (70% humidity, 360 μmol/m^−2^/s^−1^, 20–23 °C, and a 16/8 h light/dark cycle), were subjected to a 13-day drought period without water. After a 10-day drought period, physiological indicators in leaves of transgenic plants were measured, including chlorophyll, MDA, and proline levels as well as the activities of SOD, CAT, and POD [81,83,84,85]. Chlorophyll, malondialdehyde (MDA), and proline contents, as well as the activities of antioxidant enzymes SOD, CAT, and POD, were primarily determined using commercial assay kits from Solarbio (Beijing, China). Specifically, chlorophyll was measured with the Chlorophyll Assay Kit (Cat. No. BC0995), MDA with the MDA Assay Kit (Cat. No. BC6410), proline with the Proline Assay Kit (Cat. No. BC0290), SOD with the SOD Assay Kit (WST-1 method, Cat. No. BC5165), CAT with the CAT Assay Kit (Cat. No. BC0205), and POD with the POD Assay Kit (Cat. No. BC0095), following the manufacturer’s protocols. In the determination of SOD, POD, and CAT activities, the soluble protein used as the basis for enzyme assays was mainly obtained from the resulting supernatant of leaf tissue homogenates. Protein concentration was quantified using the Bradford method with bovine serum albumin (BSA) as the standard. Fresh weight (FW) of leaf samples was recorded immediately after collection. The samples were then floated in deionized water overnight to obtain turgid weight (TW), followed by drying at 85 °C for 24 h to determine dry weight (DW). Relative water content (RWC) was calculated as: RWC = [(FW − DW)/(TW − DW)] × 100%. Each experiment was conducted with three biological replicates.

For expression analysis, total RNA was extracted from the leaves of *Arabidopsis* plants after 7 d of drought stress using TRIzol reagent (TianGen) and first-strand cDNA synthesis was carried out with the Transgen EasyScript^®^ First-Strand cDNA Synthesis SuperMix following the manufacturer’s instructions. The transcription levels of *AtP5CS1*, *AtSOD* (Cu/Zn), *AtPOD1*, and *AtCAT1* were quantified by qRT-PCR, with *Actin2* employed as the internal reference gene. The transcription levels of *AtP5CS1*, *AtSOD* (Cu/Zn), *AtPOD1*, and *AtCAT1* in transgenic lines subjected to drought stress for 7 d were quantified using qRT-PCR, with *Actin 2* as the *Arabidopsis* internal reference gene. Each experiment was conducted with three biological replicates.

### 4.7. Yeast Two-Hybrid Assay

Yeast two-hybrid (Y2H) assays were performed using a Matchmaker GAL4 system (Clontech). The bait and prey constructs were co-transformed into yeast AH109. Positive transformants were verified by growth on selective media SD/-Leu/-Trp (DDO) and SD/-His/-Leu/-Trp/X-α-Gal (QDO/X).

### 4.8. Transactivation of Promoters in the Transient System

The coding sequences (CDSs) of *MsNF-YA4*, *MsNF-YB3*, and *MsNF-YC2* were inserted into the pGreenII 62-SK plasmid to serve as effector constructs, whereas a 2000 bp promoter fragment of *MsP5CS1* was cloned into the pGreenII 0800. The resulting recombinant constructs were, respectively, co-transformed into *Agrobacterium* strain GV3101: 62-SK-*MsNF-YA4*/LUC-*MsP5CS1*, 62-SK-*MsNF-YA4*/LUC-*MsSOD*, 62-SK-MsNF-YA4/LUC-*MsCAT*, and 62-SK-*MsNF-YA4*/LUC-*MsPOD*. After mixing with the infiltration buffer, the constructs were introduced into *Nicotiana* leaves. Three days post-infiltration, the detection was performed via a dual-luciferase reporter system, as outlined in previous studies [86]. Each experiment was conducted with three biological replicates.

## 5. Conclusions

This study revealed the pivotal role of the NF-Y TF MsNF-YA4 in plant drought tolerance. Whole-genome analysis of *Miscanthus* and gene expression profiling under drought condition identified MsNF-YA4 markedly upregulated in response to drought stress. The ectopic expression of *MsNF-YA4* in *Arabidopsis* regulates the expression of stress-related genes, enhances physiological traits, and significantly improves drought tolerance. Further mechanistic analysis demonstrated that MsNF-YA4/MsNF-YB3/MsNF-YC2 enhances the regulation of *Miscanthus P5CS1*, *SOD* (Cu/Zn), and *CAT1* by transactivating their promoters in a reporter system. These results offer new perspectives on the mechanisms at the molecular level governing drought tolerance in MsNF-YAs.

## Data Availability

Further inquiries can be directed to the corresponding authors.

## Supplementary Materials

The following supporting information can be downloaded at https://www.mdpi.com/article/10.3390/plants14193100/s1: Figure S1: Chromosomal location of *NF-Y* genes in *Miscanthus*; Figure S2: Analysis of conserved motifs in NF-YA proteins in *Miscanthus*; Figure S3: RWC of *Miscanthus* seedling leaves subjected to dehydration/osmotic stress; Figure S4: Relative expression levels of transgenic lines by RT-qPCR; Figure S5: Confirmation of transgenic *MsNF-YA4 Arabidopsis* lines through RT-PCR; Figure S6: MsNF-YA4 protein network interaction using *Arabidopsis* model; Figure S7: Screening of MsNF-YA4 interacting proteins; Figure S8: Analysis of CCAAT-box *cis*-acting element in the promoters of *MsP5CS1*, *MsSOD*(Cu/Zn), *MsPOD1*, and *MsCAT1*. Table S1: Physical locations and properties of *MsNF-YA* genes in *Miscanthus*; Table S2: Ka/Ks ratio of paralogous pairs of NF-YA genes in Miscanthus; Table S3: Candidate protein interacting with MsNF-YA4; Table S4: Primer pairs used in the present study.
