# Supplementary material for: Comprehensive Analysis of Miscanthus NF-YA Genes Reveals Potential Involvement in Drought Stress Adaptation
Source: Plants (Basel). 2025 Oct 8;14(19):3100. doi: 10.3390/plants14193100 (PMC12526792; doi:10.3390/plants14193100)
Supplement: Supplementary file 1 [file plants-14-03100-s001.zip › plants-3803638-Supplementary.pdf]

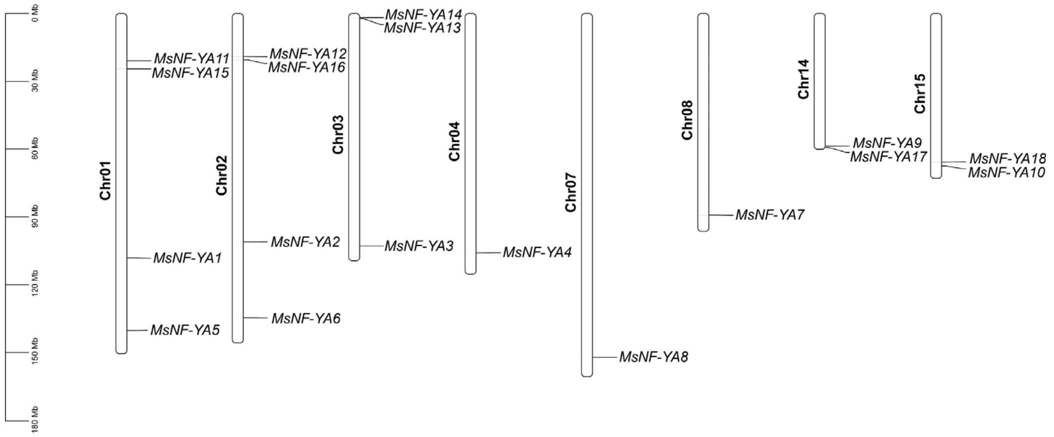

Figure S1. Chromosomal location of *NF-Y* genes in *Miscanthus*.

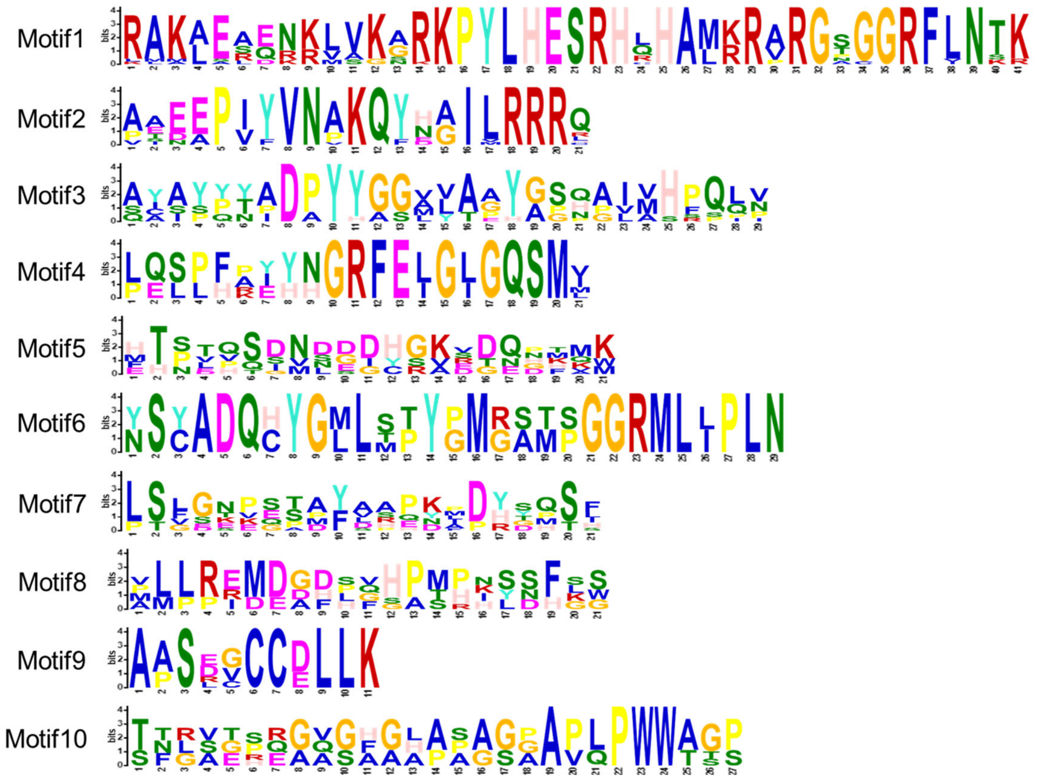

Figure S2. Analysis of conserved motifs in NF-YA proteins in *Miscanthus*.

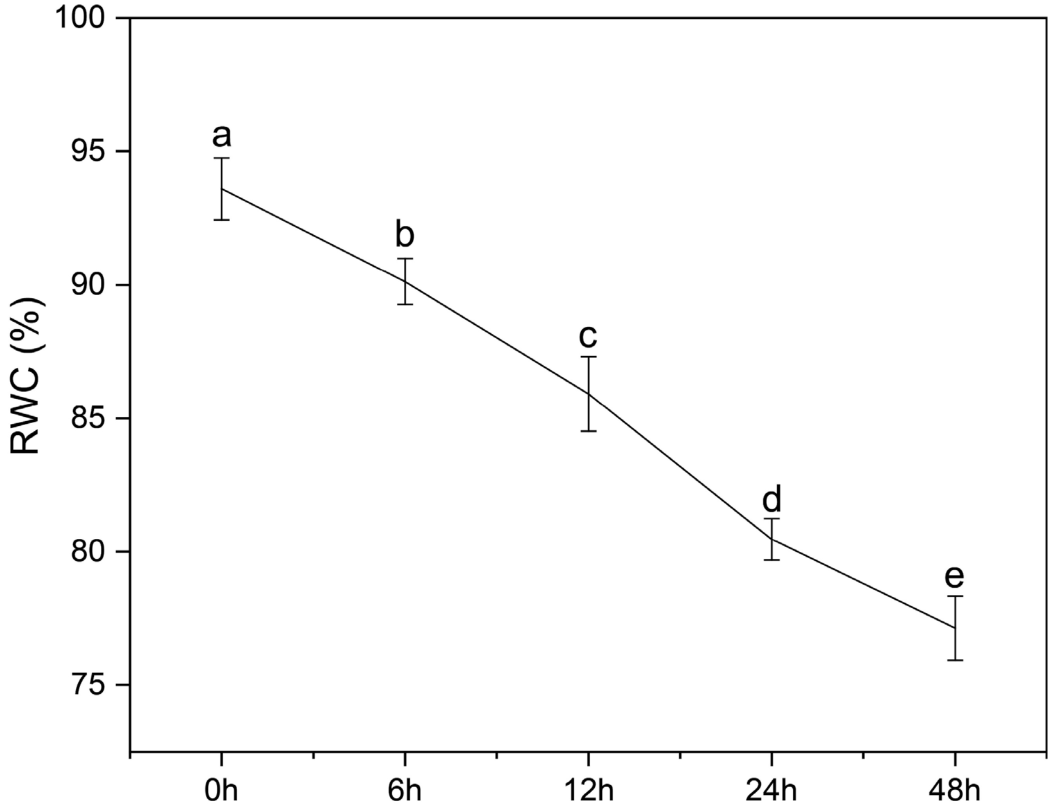

Figure S3. RWC of *Miscanthus* seedling leaves subjected to dehydration/osmotic stress. According to Tukey’s test, differences were assessed using one-way analysis of variance (ANOVA) at $P < 0.05$.

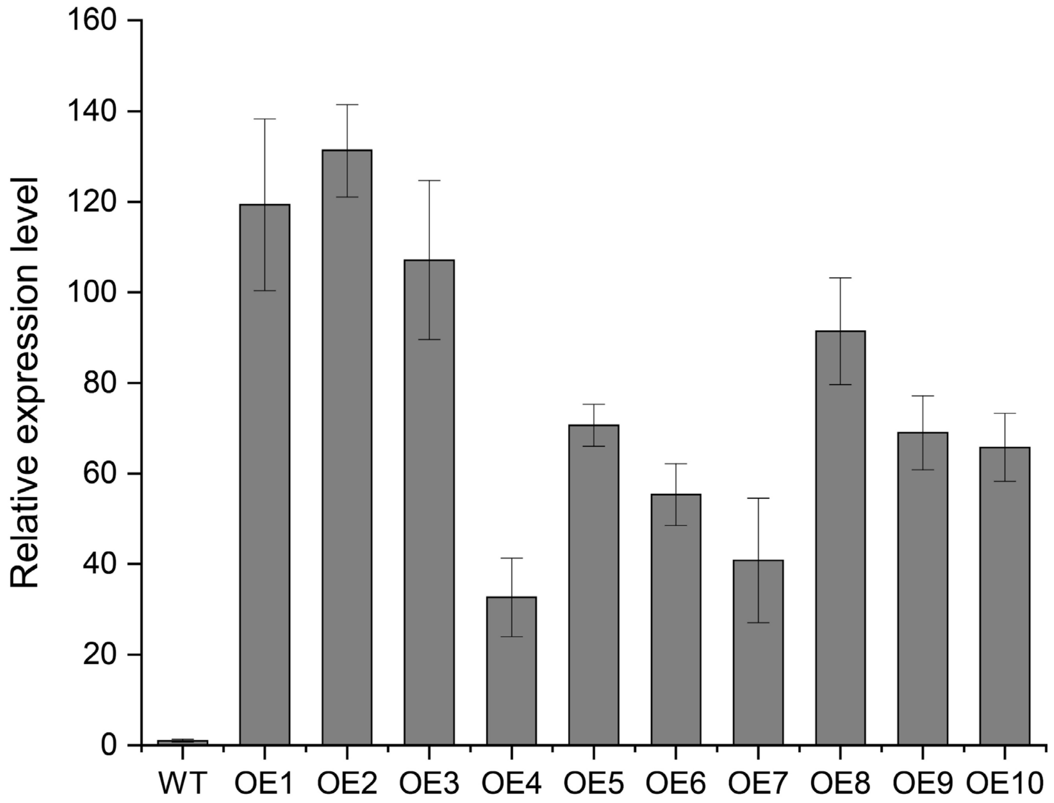

Figure S4. Relative expression levels of transgenic lines by RT-qPCR.

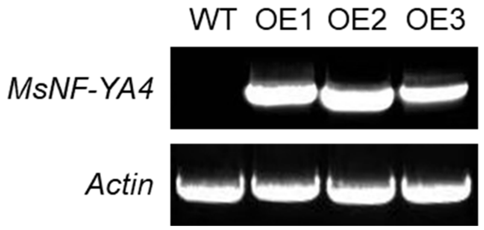

Figure S5. Confirmation of transgenic *MsNF-YA4 Arabidopsis* lines through RT-PCR.

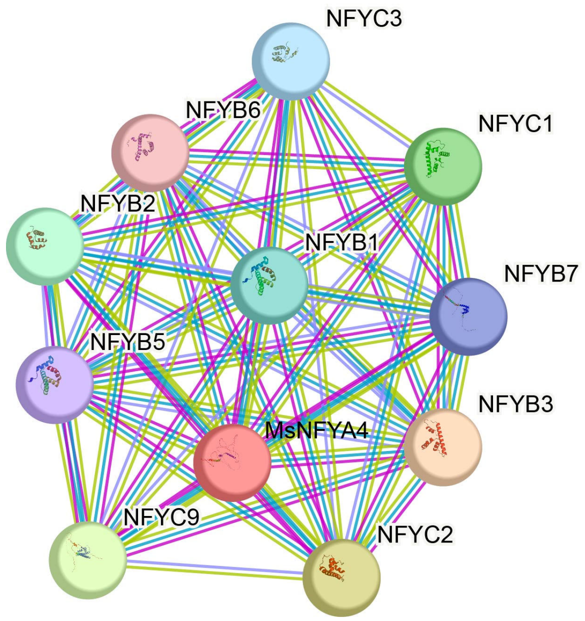

Figure S6. MsNF-YA4 protein network interaction using *Arabidopsis* model.

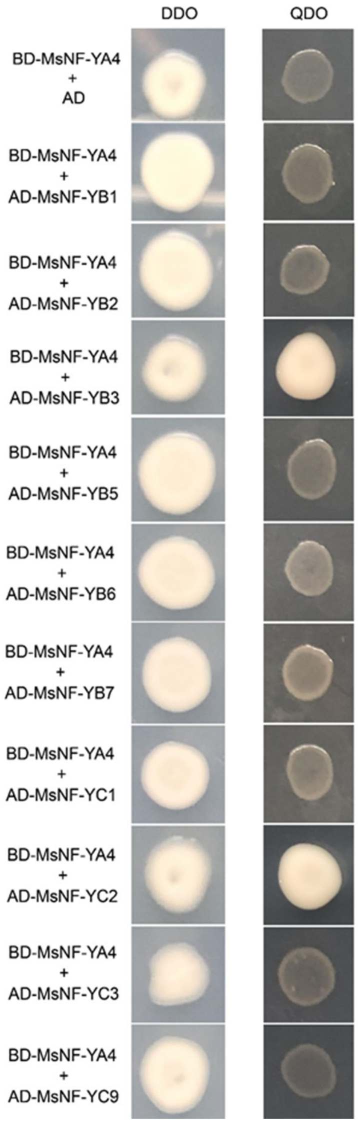

Figure S7. Screening of MsNF-YA4 interacting proteins. Yeast point-to-point detection between MsNF-YA4 and interacting candidates. Positive transformants were verified by growth on selective media SD/-Leu/-Trp (DDO) and SD/-His/-Leu/-Trp/X-α-Gal (QDO/X).

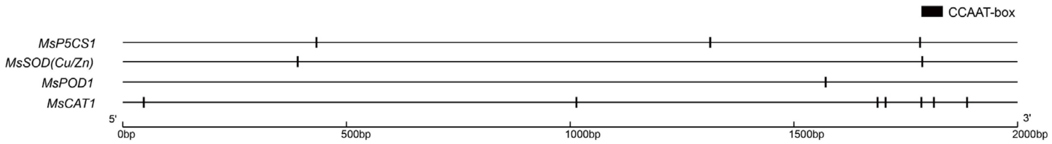

Figure S8. Analysis of CCAAT-box *cis*-acting element in the promoters of *MsP5CS1*, *MsSOD(Cu/Zn)*, *MsPOD1*, and *MsCAT1*.
